# Preparation and evaluation of poly(2-hydroxyethyl aspartamide)-hexadecylamine-iron oxide for MR imaging of lymph nodes

**DOI:** 10.1186/1556-276X-9-38

**Published:** 2014-01-18

**Authors:** Dae Chul Ha, Ha Young Lee, Yeo Won Son, Soon Hong Yuk, Youn Woong Choi, Byung Kook Kwak, Byung Cheol Shin, Cheong-Weon Cho, Sun Hang Cho

**Affiliations:** 1 College of Pharmacy, Chungnam National University, 99, Daehak-ro, Yuseong-gu, Daejeon 305-764, Republic of Korea; 2 Division of Drug Discovery Research, Research Center for Medicinal Chemistry, Korean Research Institute of Chemical Technology (KRICT), 100 Jang-dong, Yuseong-gu, Daejeon 305-600, Republic of Korea; 3 University of Science and Technology, 113 Gwahangno, Yuseong-gu, Daejeon 305-333, Republic of Korea; 4 College of Pharmacy, Korea University, 2511 Sejongro, Sejong 339-700, Republic of Korea; 5 Korea United Pharmaceuticals, Inc., 25-23 Nojanggongdan-gil, Jeondong-myeon, Sejong 339-841, Republic of Korea; 6 Chung-Ang University, 102 Heukseok-ro, Dongjak-gu, Seoul 156-755, Republic of Korea

**Keywords:** Magnetic resonance imaging, Lymph node, Ultrasuperparamagnetic iron oxide, Nanoparticles

## Abstract

The purpose of this study was to synthesize biocompatible poly(2-hydroxyethyl aspartamide)–C_16_-iron oxide (PHEA-C_16_-iron oxide) nanoparticles and to evaluate their efficacy as a contrast agent for magnetic resonance imaging of lymph nodes. The PHEA-C_16_-iron oxide nanoparticles were synthesized by coprecipitation method. The core size of the PHEA-C_16_-iron oxide nanoparticles was about 5 to 7 nm, and the overall size of the nanoparticles was around 20, 60, and 150 nm in aqueous solution. The size of the nanoparticles was controlled by the amount of C_16_. The 3.0-T MRI signal intensity of a rabbit lymph node was effectively reduced after intravenous administration of PHEA-C_16_-iron oxide with the size of 20 nm. The *in vitro* and *in vivo* toxicity tests revealed the high biocompatibility of PHEA-C_16_-iron oxide nanoparticles. Therefore, PHEA-C_16_-iron oxide nanoparticles with 20-nm size can be potentially useful as T2-weighted MR imaging contrast agents for the detection of lymph nodes.

## Background

Accurate diagnosis of lymph node metastasis in various cancer patients is very important as it is one of the most important factors for the choice of preoperative chemoradiotherapy, surgical treatment, and patient prognosis [1]. Recently, magnetic resonance (MR) imaging with ultrasmall superparamagnetic iron oxide (USPIO) has gained approval as a noninvasive method for the detection of lymph node metastases in several tumors [2,3]. MR provides images with excellent anatomical detail but, at the same time, is relatively insensitive to lymph node metastases due to the limited sensitivity of current node size criteria in differentiating benign from malignant nodes. However, the MR results can be improved by using a superparamagnetic contrast agent such as USPIO [4,5]. USPIO acts as a negative contrast agent, and therefore, normal functioning lymph nodes can be distinguished from lymph node metastases on the basis of magnetic resonance signal characteristics, independent of nodal size [6-9]. USPIO nanoparticles have offered new potential for early detection of lymph nodes and their metastases using magnetic resonance imaging (MRI). These nanoparticles are taken up by macrophages in normal lymph nodes and show signal reduction (e.g., by susceptibility-based relaxation). Because less nanoparticle uptake is seen in metastatic nodes, these nodes may be overlooked as a result of the minimal signal change in the occupying mass. Fortunately, among various MR protocols that show the lymph nodes, susceptibility base sequences are very sensitive to small magnetic changes inside the nodes [10]. Some USPIO particles (mean diameter less than 50 nm) are used as MRI contrast agents (e.g., Sinerem®, European Medicines Agency, Canary Wharf, London; Combidex®, Radboud University Nijmegen Medical Centre, Nijmegen, Netherlands; Clariscan™, Nycomed, Drammensveien, Asker, Norway) to differentiate metastatic nodes from lymph nodes. These USPIOs are composed of iron oxide nanoparticles coated with polymers such as low molecular weight dextran and polyethyleneglycol (PEG).

In the present study, we used an amphiphilic poly(2-hydroxyethyl aspartamide) (PHEA) graft copolymer, and not a block copolymer, to form biocompatible USPIO (less than 30 nm in diameter). PHEA, which is a poly(amino acid) derivative, was used to coat the iron oxide (Fe_3_O_4_) nanoparticles for MRI applications. PHEA is a synthetic polymer having a protein-like structure, obtained by the reaction of ethanolamine with polysuccinimide (PSI), which is prepared by thermal polycondensation of d,l-aspartic acid. PHEA has good biopharmaceutical properties as drug carrier such as high water solubility, multi-functionality, absence of toxicity, antigenicity, immunogenicity, and low cost of production [11-17]. Hydrophobic side chain was grafted to the PHEA backbone to aid in good solubility of hydrophobic Fe_3_O_4_ nanoparticles in aqueous phases. Hexadecyl alkyl groups permit hydrophobic interaction with ligands on Fe_3_O_4_ nanoparticles, and hydrophobic van der Waals interaction affords good stability in aqueous solution [18].

In this paper, we synthesized amphiphilic graft derivatives of PHEA by the introduction of hydrophobic hexadecylamine (C_16_) as a linker of iron oxide. We evaluated the feasibility of using PHEA-C_16_-iron oxide nanoparticles as MRI contrast agent for the detection of lymph nodes and performed *in vitro* and *in vivo* toxicology studies.

## Methods

### Materials

Polysuccinimide (2,000 ~ 3,000 g/mol) was purchased from Baypure, Bayer Chemicals AG, Leverkusen, Germany. 1-Hexadecylamine (C_16_-NH_2_), ethanolamine, ammonium hydroxide (NH_3_ content 28 % ~ 30 %), ferrous chloride tetrahydrate (FeCl_2_ · 4H_2_O), ferric chloride hexahydrate (FeCl_3_ · 6H_2_O) were purchased from Aldrich Chemical Company, Inc., Milwaukee, WI, USA. *N*,*N*-dimethylformamide (DMF) was purchased from Junsei Chemical Co., Ltd., Tokyo, Japan. Hallow fiber filter membrane (dialysis with tangential flow separation module) and peristatic pump for the purification of large amounts of contrast agent were purchased from KD Scientific, Holliston, MA, USA. Freeze dryer Bondiro and deep freezer Gudero were purchased from Ilshin, Daejeon, South Korea. Pure 18.2 MΩ cm distilled water was used by Milli-Q, Millipore, Molsheim, France. All the other reagents were commercially available and used without further purification. A commercial contrast agent, Resovist® 1.4 ml (SH U 555 A) as a control for comparison with experiment was purchased from Schering Bayer AG, Berlin, Germany.

### Synthesis of PHEA-C_16_-iron oxide

Different amounts of C_16_ (3.26, 6.51, and 13.02 g) in DMF (15, 30, and 60 ml) were added dropwise to a solution of PSI (15 g, 1.5 × 10^−3^ mol) in DMF (100 ml). The mixture was stirred at 70°C under a nitrogen atmosphere. After 1 h, ethanolamine (7.49 ml, 0.125 mol) was added dropwise to the reaction solution. The mixture was stirred at room temperature for 24 h, under a nitrogen atmosphere. The reaction solution was precipitated and filtered with ethyl ether for the removal of DMF and unreacted materials. The filtered PHEA-C_16_ was washed with ethyl ether and dried under vacuum at 60°C. PHEA-C_16_ (1.16 g, 2 × 10^−4^ mol), FeCl_2_ · 4H_2_O (0.0794 g, 4 × 10^−4^ mol), and FeCl_3_ · 6H_2_O (0.1584 g, 6 × 10^−4^ mol) were dissolved in distilled water (40 ml). The resulting mixture was stirred at 80°C for 1 h under vigorous mechanical stirring, and ammonia solution (3 ml) was added dropwise to the reaction solution. During this process, the initial orange color of the solution gradually turned into a brownish black colloidal solution. The colloidal solution was cooled to room temperature for 10 min, and distilled water (80 ml) was added to the brownish black colloidal solution. This solution was centrifuged at 6,000 rpm for 15 min. The upper-layer solution of the unreacted polymer was removed from the PHEA-C_16_-iron oxide, with the pH adjusted, and concentrated using hollow fiber. And, the dialyzed colloidal solution was freeze-dried.

### Characterization analysis

The results of polymerization were confirmed by ^1^H NMR (300 and 500 MHz, Bruker, Billerica, MA, USA). Dimethyl sulfoxide (DMSO, Aldrich) was used as a solvent. Particle size distribution of the PHEA-C_16_-iron oxide nanoparticles in the colloidal was analyzed using an electrophoresis light scattering (ELS 8000, OTSUKA Electronics Co., Ltd., Osaka, Japan). The size and morphology of the PHEA-C_16_-iron oxide nanoparticles were examined using a transmission electron microscope (TEM; JEM-2010, JEOL, Tokyo, Japan). The powder after being dissolved in ethanol was drop casted onto a 300-mesh carbon-coated copper grid, and the grid was air-dried at room temperature before viewing under the microscope. The magnetic properties of the PHEA-C_16_-iron oxide nanoparticles were carried out at room temperature with */H/* ≤20 kOe using Quantum Design MPMS 5 SQUID magnetometer (San Diego, CA, USA).

### *In vitro* MRI test

To confirm the feasibility of the PHEA-C_16_-iron oxide nanoparticles as an MRI contrast agent, we first prepared a ferrofluid phantom consisting of PHEA-C_16_-iron oxide nanoparticles and Resovist® with Fe concentrations varying from 9 to 1,250 μM in deionized water. Every sample was filled into an arrangement of 2-ml tubes without air in a plastic rack. The tubes containing the samples were embedded in a phantom which consisted of tanks filled with water to obtain appropriate image [19]. MRI was performed using a 3.0-T MRI system (Intera Achieva 3.0 T, Philips Medical Systems, Philips Co., Best, The Netherlands). The T2-weighted image of the phantom was obtained with a turbo spin echo technique. The sequence parameters were 4,341 ms of repetition time (TR), 79.4 ms of echo time (TE), 1-mm thickness, and 256-mm field of view (FOV).

### *In vivo* MRI test

To observe the *in vivo* MRI effect of lymph node, T2-weighted MRI was performed in normal healthy New Zealand White rabbits weighing 3.3 kg. The rabbits were anesthetized with an injection of pentobarbital (1 M). The PHEA-C_16_-iron oxide (0.45 mmol Fe, 25.13 mg Fe) was administered into a marginal vein of the rabbit's ear through the catheter. The T2-weighted MRI images were obtained before administration and 20 min after administration of the PHEA-C_16_-iron oxide nanoparticles. The T2-weighted MR imaging of the lymph node was performed with a turbo spin echo technique. The sequence parameters were 5,383 ms of TR, 79.4 ms of TE, 1-mm thickness, and 112-mm FOV. The rabbit was sacrificed immediately after MR imaging test for the confirmation of PHEA-C_16_-iron oxide nanoparticles taken up by lymph node. The specimens was fixed in 10% formalin for 24 h and embedded in paraffin. Prussian blue staining was performed on 5-μm thick sections: the preparations were treated with a mixture of equal parts of 20% hydrochloric acid and 10% potassium ferrocyanide solution for 20 min and counterstained with nuclear fast red for 5 min. The principle of this method is that the ferric iron (Fe^3+^) in the tissue combines with the ferrocyanide and results in the formation of a bright blue pigment called Prussian blue.

### *In vitro* and *in vivo* toxicity tests

#### A single-dose intravenous toxicity study of PHEA-C_16_-iron oxide in Sprague–Dawley rats

This study was carried out to evaluate the toxicity of PHEA-C_16_-iron oxide in Sprague–Dawley rats (8 weeks old, Koatech, Seoul, South Korea) after a single intravenous administration. PHEA-C_16_-iron oxide was administered intravenously at dose levels of 0 (vehicle control), 800, 1,200, and 1,800 mg/kg. Each group consisted of five rats of both sexes. After the administration, the mortality rate, clinical signs, body weights, and gross necropsy findings were compared with those of the vehicle control group. All experiments were performed at the preclinical research center of ChemOn, Inc., a nationally recognized testing laboratory recognized by Korea Food and Drug Administration (KFDA), according to the guidelines of GLP: the standards of toxicity study for medicinal products (notification no. 2005–60 (October 21, 2005, KFDA)) and good laboratory practice regulation for nonclinical laboratory studies (notification no. 2005–79 (December 21, 2005, KFDA)).

#### Micronucleus test of PHEA-C_16_-iron oxide in bone marrow cells of female ICR mice (intravenous study)

A micronucleus test of PHEA-C_16_-iron oxide was conducted using female imprinting control region (ICR) strain-specific pathogen-free (SPF) mice. The PHEA-C_16_-iron oxide was dissolved in sterilized distilled water. Seven-week-old female ICR mice were intravenously administered with PHEA-C_16_-iron oxide for two consecutive days at doses of 0, 125, 250, and 500 mg/kg/day. Six mice were assigned per dose group. The positive control (cyclophosphhamide∙H_2_O, 70 mg/kg) was given once intraperitoneally on the day of second administration. All animals were sacrificed about 24 h after the second administration, and bone marrow preparations were made.

#### Bacterial reverse mutation test of PHEA-C_16_-iron oxide

Bacterial reverse mutation test of PHEA-C_16_-iron oxide was evaluated for its potential effect to induce reverse mutation in the histidine auxotroph strains of *Salmonella typhimurium* TA 100, TA 1535, TA 98, and TA 1537, and atryptophan auxotroph strain of *Escherichia coli* WP2 *uvrA*. PHEA-C_16_-iron oxide for treatment was dissolved in sterilized distilled water, filtered with a syringe filter (pore size 0.2 μm), and serial dilutions were made. All bacterial strains were exposed to PHEA-C_16_-iron oxide in the presence and absence of exogenous metabolic activation system. The metabolic activation system was prepared with Aroclor 1254-induced rat liver homogenate (S-9) and cofactor. The dose levels of the PHEA-C_16_-iron oxide were 62, 185, 556, 1,667, and 5,000 μg/plate for all strains. For all strains, corresponding vehicle control and positive control groups were also included. Three plates were used per dose, and tester strains were exposed to the PHEA-C_16_-iron oxide by direct plate incorporation method. The plates were incubated for about 48 h after treatment, and colonies were counted.

#### In vitro *mammalian chromosome aberration test of PHEA-C*_*16*_*-iron oxide in cultured Chinese hamster lung cells*

This study was carried out to evaluate the mutagenic potential of PHEA-C_16_-iron oxide in terms of clastogenicity using cultured Chinese hamster lung (CHL) cells in the presence (+S) and absence (−S) of metabolic activation system. The metabolic activation system was prepared with Aroclor 1254-induced rat liver homogenate and cofactor. The PHEA-C_16_-iron oxide was suspended in complete culture medium (minimum essential medium), and serial dilutions were made for treatment. The highest dose for each treatment series was determined based on the relative cell count (RCC) as a cytotoxicity index. The doses are 138, 275, 550, and 600 μg/ml (6 h); 10, 20, 40, and 50 μg/ml (6 h); and 10, 20, 30, 40 μg/ml (24 h) with appropriate negative and positive controls to evaluate the mutagenic potential of PHEA-C_16_-iron oxide in presence (+S), absence (−S), and absence (−S) of metabolic activation system, respectively.

All the experiments were performed in duplicate for each dose. Rapidly growing cell cultures were trypsinized, and three series of 25-cm^2^ culture flasks (Falcon, Corning, Inc., Tewksbury MA, USA) were seeded with 6 × 10^4^ cells, each in 5-ml medium, and incubated for 3 days before the chemical treatment. The mitotic cells of each flask were harvested 24 h after the start of treatment, and chromosome preparations were made. A hundred metaphases per culture (200 metaphases per dose) were analyzed from a selected slide in each culture.

All tests were performed according to the guidelines of GLP: the standards of toxicity study for medicinal products (notification no. 2005–60 (October 21, 2005, KFDA)), good laboratory practice regulation for nonclinical laboratory studies (notification no. 2005–79 (December 21, 2005, KFDA)), and OECD Guideline for the Testing of Chemicals (TG 473 (1997) ‘*In vitro* Mammalian Chromosomal Aberration Test’).

## Results

### Synthesis and characterization of PHEA-C_16_-iron oxide

**Figure 1:**
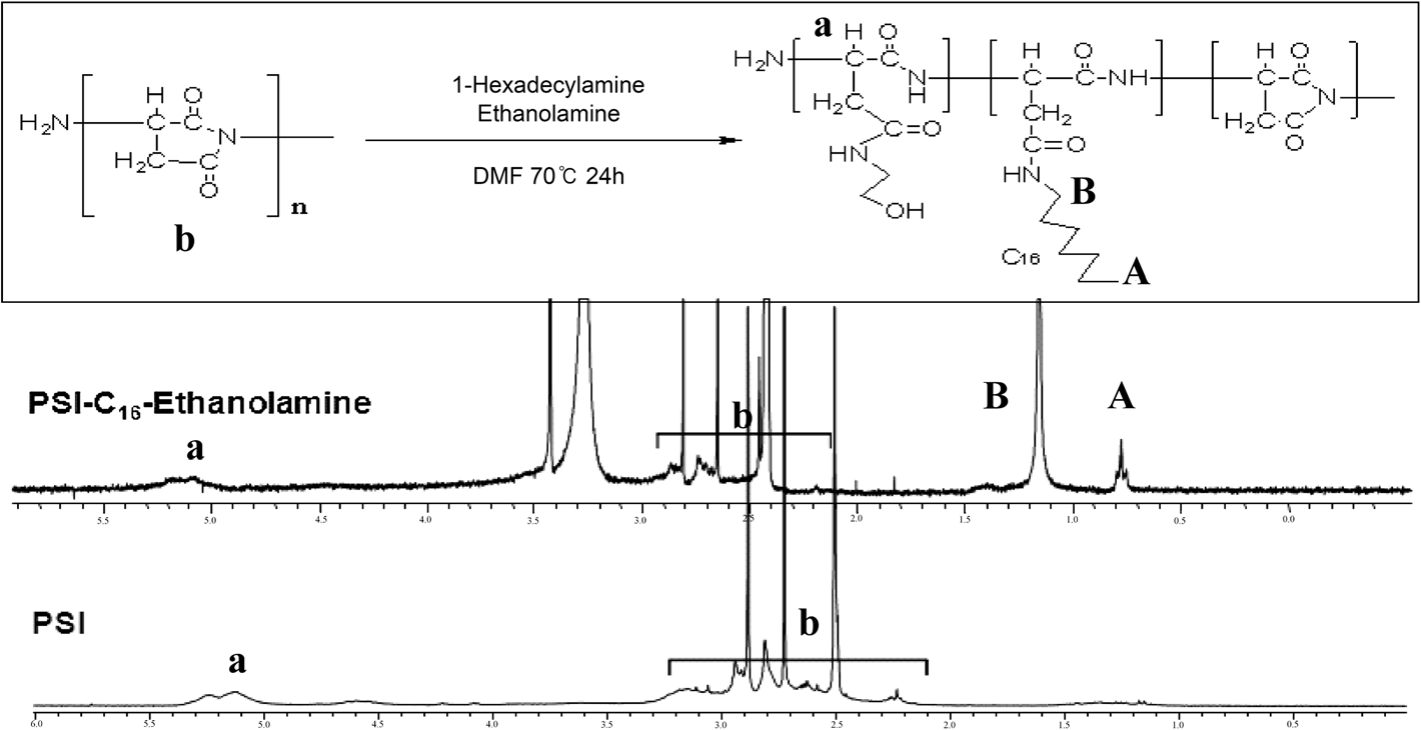
**The overall synthetic scheme of PHEA-C**_**16**_**and**^**1**^**H NMR spectra of PSI, PHEA-C**_**16**_**.**

To prepare the PHEA-C_16_-coated iron oxide nanoparticles as a contrast agent, PHEA-C_16_ was synthesized by aminolysis of PSI with ethanol amine and hexadecylamine. The overall synthetic route of PHEA-C_16_ and ^1^H NMR of PSI and PHEA-C_16_ are shown in Figure 1. In PSI, the signals at 5.04 ppm were assigned to the methylene proton (a) of the repeating succinimide unit, and those at 2.5 to 3.5 ppm were assigned to methylene proton (b). The degree of substitution (DS) of C_16_ in the grafted PHEA polymer was calculated by comparing the integral of the CH_2_ peak at *δ* 1.3 of hexadecylamine to the integral of the peak at *δ* 5.04 assigned to the protons that belong to the PSI unit. Hexadecyl alkyl groups interact with iron oxide nanoparticles through hydrophobic van der Waals interaction, which lead to a formation of a spherical-shaped structure [18]. Hydrophobic Fe_3_O_4_ nanoparticles were synthesized by a typical coprecipitation method. The particle sizes were controlled by varying the degree of substitution of the hydrophobic hexadecylamine groups. With the increase of DS of C_16_, the mean particle sizes decreased, as presented in Table 1.

**Table 1:** **Particle size with various amounts of C**_**16**_

**Overall particle size of PHEA-C**_**16**_**-iron oxide (nm)**	**C**_**16**_**DD (mol%)**
20	35
60	25
150	15

### Characterization analysis of iron oxide nanoparticles

**Figure 2:**
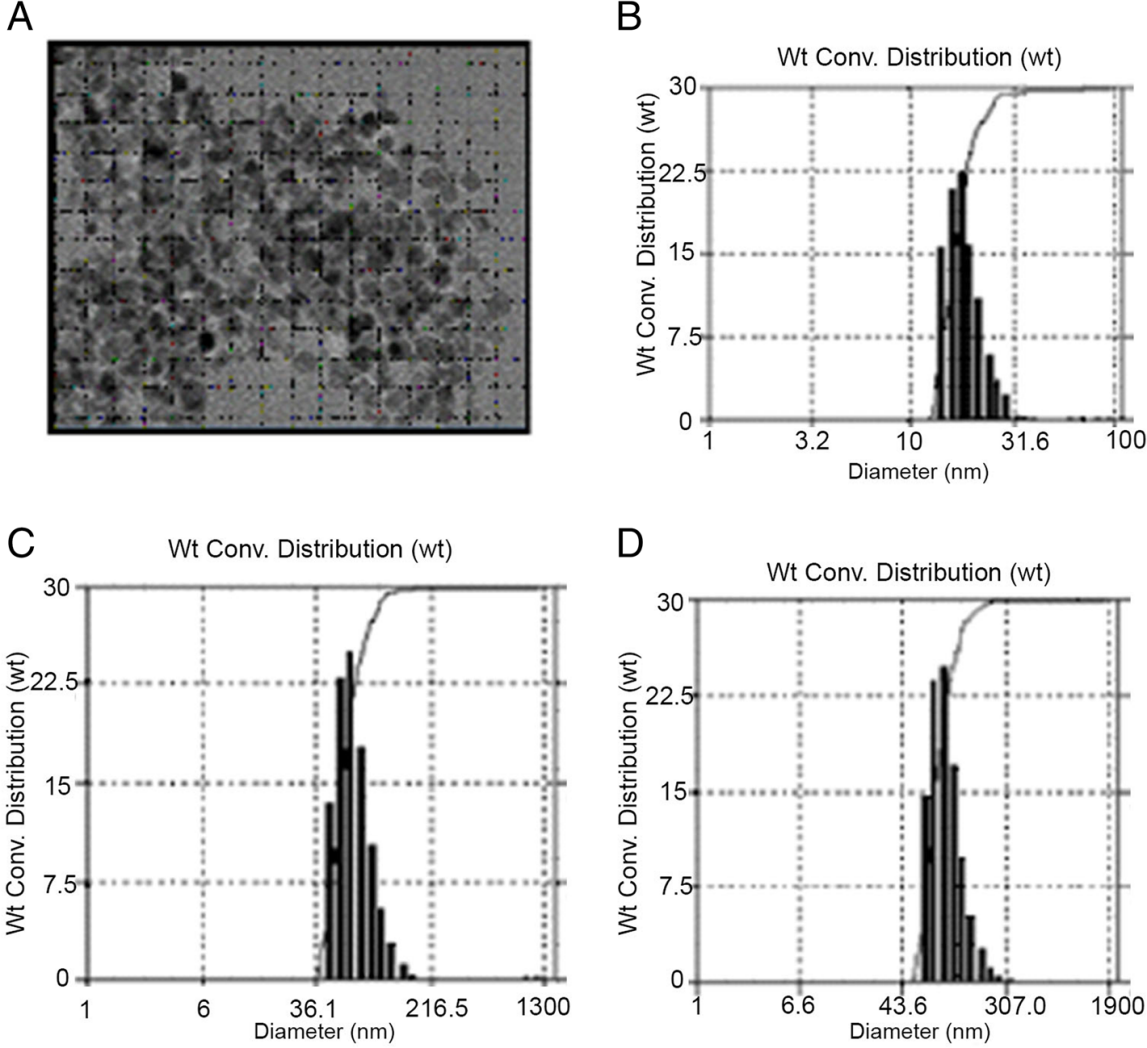
**TEM image and size distribution.(A)** TEM image and size distribution of **(B)** 20-nm, **(C)** 60-nm, and **(D)** 150-nm PHEA-C_16_-iron oxide nanoparticles.

Figure 2 is the TEM image and DLS data of the PHEA-C_16_-iron oxide nanoparticles. The TEM image shows that the diameter of the iron oxide core is 5 to 7 nm as illustrated in Figure 2A. In the aqueous solution, the colloidal nanoparticles have a hydrodynamic diameter of about 20, 60, and 150 nm with various amounts of C_16_ by dynamic light scattering (DLS) measurements (Figure 2B,C,D).

**Figure 3:**
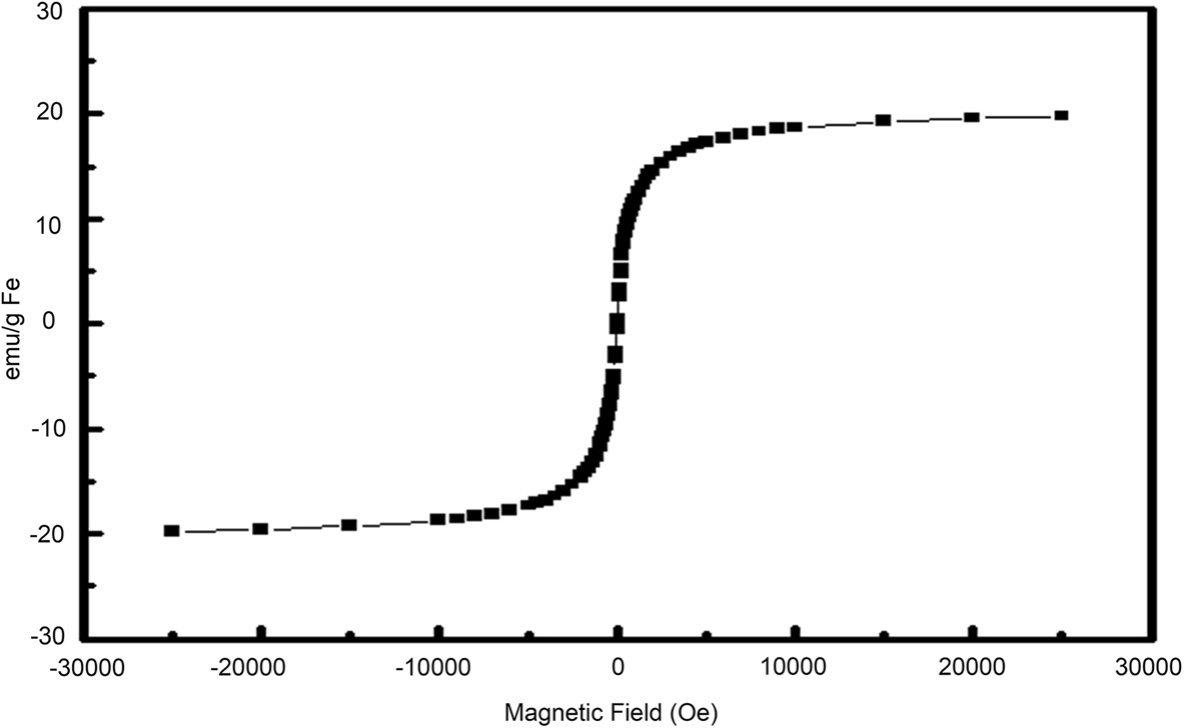
**Magnetization curve of the PHEA-C**_**16**_**-iron oxide nanoparticles with 20 nm at room temperature.**

The hysteresis loop of the PHEA-C_16_-iron oxide nanoparticles shown in Figure 3 had no coercive force, showing superparamagnetic behavior and a high magnetic moment in a high magnetic field (generally, about 5 to about 90 emu/g of metal oxide). The saturation magnetization of the PHEA-C_16_-coated iron oxide nanoparticles was 20 emu/g Fe.

### *In vitro* MRI test

**Figure 4:**
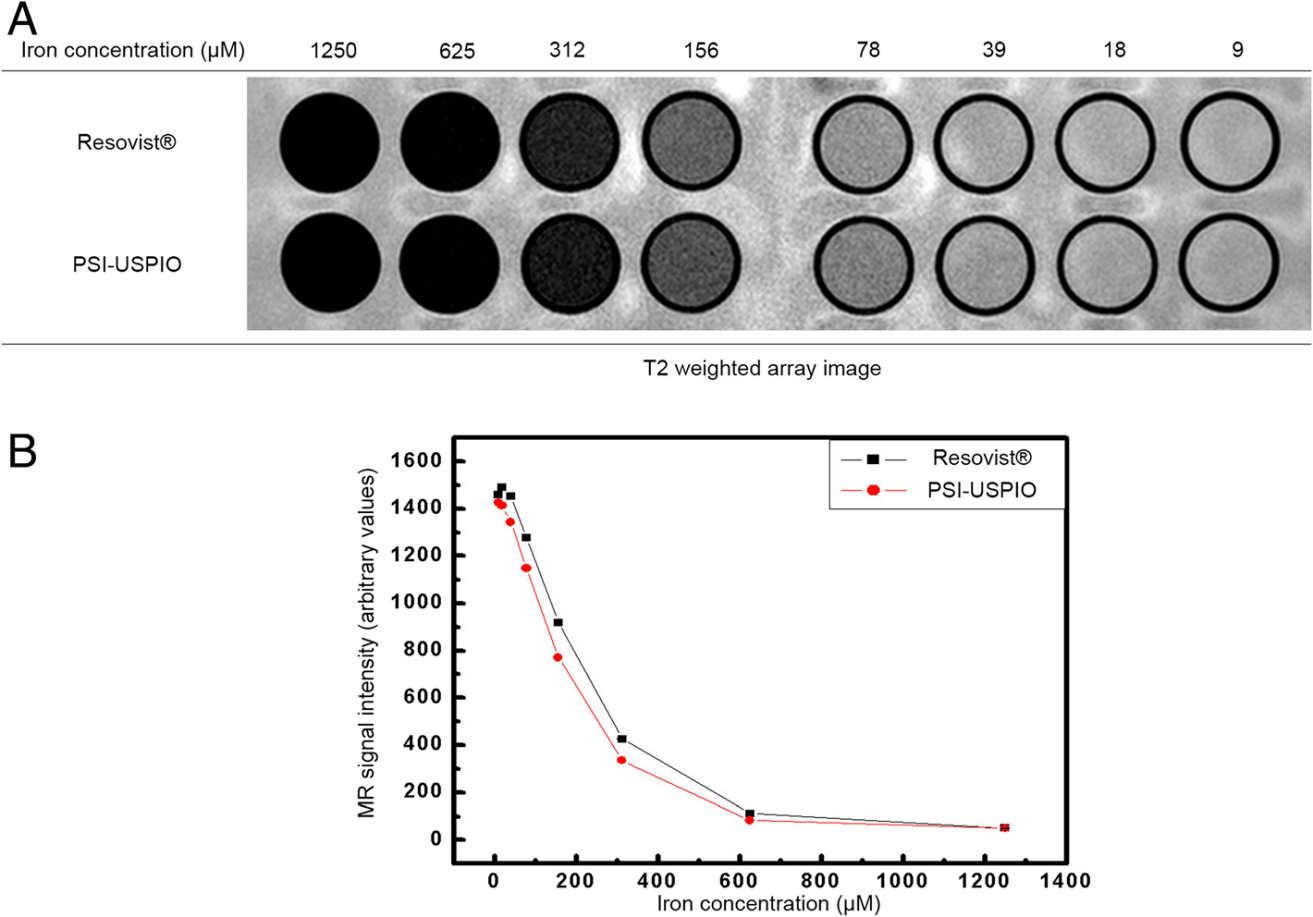
**Images of the synthesized PHEA-C**_**16**_**-iron oxide colloids and Resovist®, and signal intensity values.(A)** Phantom images acquired from T2-spin-eco-weighed MR images at different iron concentrations. **(B)** The T2 MR signal intensity is affected by the iron concentrations of Resovist® and the PHEA-C_16_-iron oxide.

T2-weighted MRI was obtained with a 3.0-T MRI system (Intera Achieva 3.0 T, Philips Medical Systems, Philips Co.) for the comparison of the MR contrast effect of the phantom made of the synthesized PHEA-C_16_-iron oxide colloids and Resovist®. Figure 4A shows the images of the synthesized PHEA-C_16_-iron oxide colloids and Resovist® in the same concentration gradient in distilled water. Figure 4B shows the signal intensity values converted by the image analysis tool for quantitative measurement. The results indicate that the PHEA-C_16_-iron oxide is slightly better than Resovist® as a T2 negative contrast agent for MRI.

### *In vivo* MRI test

**Figure 5:**
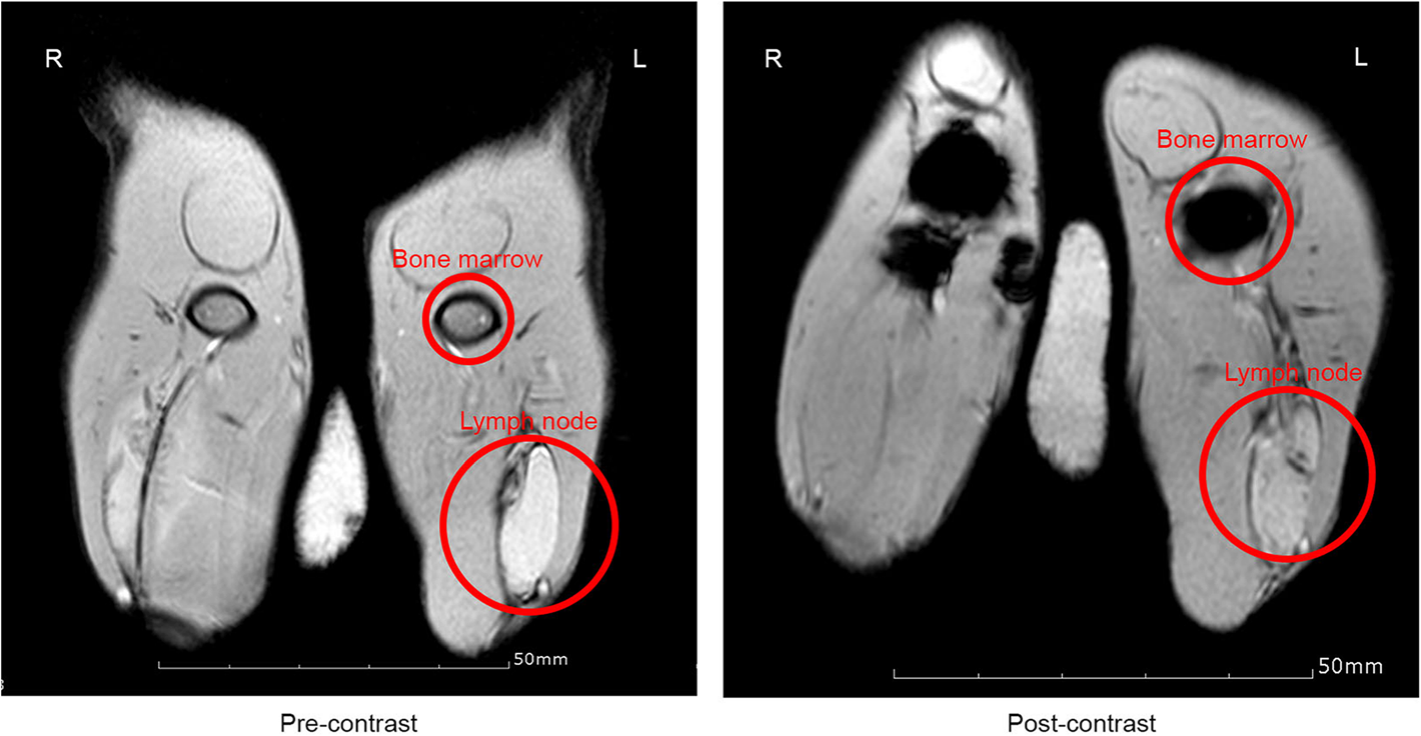
**T2-weighted MRI images of the PHEA-C**_**16**_**-iron oxide nanoparticles into a rabbit.** Lymph node and bone marrow MR images of a rabbit before (left) and after (right) the injection of 20-nm PHEA-C_16_-iron oxide nanoparticles.

**Figure 6:**
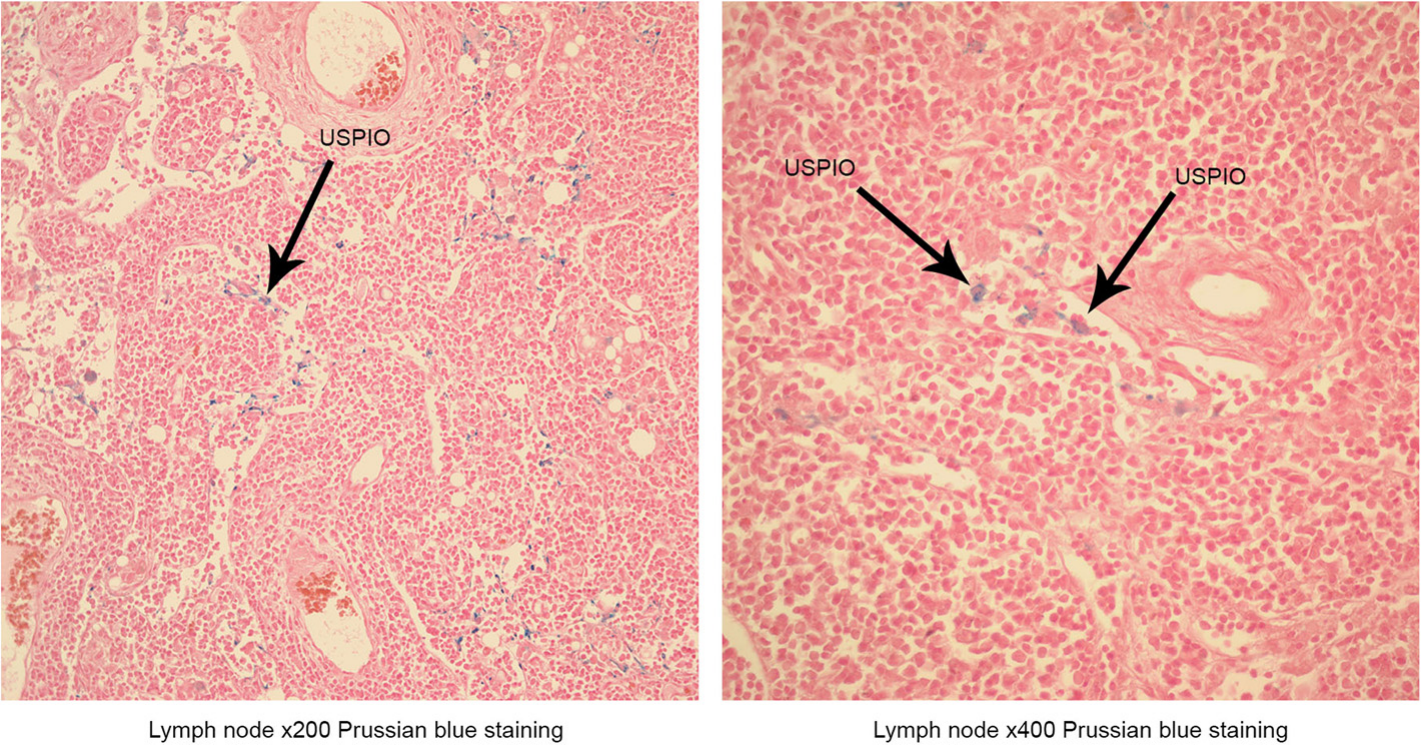
**Prussian blue staining.** Prussian blue-stained iron taken up by the lymph node of a rabbit after injection of 20-nm PHEA-C_16_-iron oxide. Lymph node at × 200 (left) and × 400 magnifications.

The PHEA-C_16_-iron oxide nanoparticles were injected intravenously into a marginal vein of the rabbit's ear through the catheter to obtain T2-weighted MRI images. Figure 5 shows the T2-weighted MRI images before and after the injection of the PHEA-C_16_-iron oxide nanoparticles into a rabbit. It shows a profound negative enhancement of the bone marrow and lymph node after the injection of the PHEA-C_16_-iron oxide nanoparticles. From the results, we confirmed that the synthesized PHEA-C_16_-iron oxide significantly improved the detection of the bone marrow and lymph node by MRI. Figure 6 shows the uptake of the PHEA-C_16_-iron oxide nanoparticles by the lymph node of rabbit on Prussian blue staining 20 min after the injection of PHEA-C_16_-iron oxide.

### *In vitro* and *in vivo* toxicity tests

#### A single-dose intravenous toxicity study of PHEA-C_16_-iron oxide in Sprague–Dawley rats

All male and female animals in the 1,800-mg/kg treatment group were observed dead, and one dead female animal was found in the 1,200-mg/kg treatment group. The clinical signs included abnormalities attributable to the PHEA-C_16_-iron oxide such as hematuria, loss of tail, soft stool, reddish tear, hypothermia, inanimation, and death. Based on the aforementioned results, following the single-dose intravenous administration of PHEA-C_16_-iron oxide in Sprague–Dawley rats, the lethal dose 50 (LD50) was 1,494 mg/kg Fe for males and 1,300 mg/kg Fe for females. A 95% confidence interval was not calculated.

#### Micronucleus test of PHEA-C_16_-iron oxide in bone marrow cells of female ICR mice (intravenous study)

There was no statistically significant increase in the frequencies of MNPCEs among 2,000 PCEs in any dose level of PHEA-C_16_-iron oxide. The ratio of PCE to total erythrocytes (PCE/PCE + NCE) was 0.36 in all groups treated with PHEA-C_16_-iron oxide, and there was no statistically significant difference at any dose levels of PHEA-C_16_-iron oxide when compared with the vehicle controls. No mortality was observed at any of the dose levels, and there were no abnormal clinical signs attributed to the administration of PHEA-C_16_-iron oxide. There was no significant change in body weights as well. Therefore, PHEA-C_16_-iron oxide did not induce micronucleus in the bone marrow cells of ICR mice used in the present study.

#### Bacterial reverse mutation test of PHEA-C_16_-iron oxide

There was no microbial colony in any of the plates for sterility check of high-dose PHEA-C_16_-iron oxide and S-9 mix. The viable cell counts of tester strains were 0.71 × 10^9^ to 1.65 × 10^9^ CFU/ml for TA strains, and 2.31 × 109 CFU/ml for WP2 *uvrA*. It showed that appropriate numbers of bacteria were treated. There was no precipitation of the PHEA-C_16_-iron oxide in the top agar. There was neither significant increase nor growth inhibition of colony in all PHEA-C_16_-iron oxide-treated plates in all strains. In all positive control groups, positive response was obtained. It was concluded that the PHEA-C_16_-iron oxide was not able to induce reverse mutation in the tester strains used in this study.

#### In vitro *mammalian chromosome aberration test of PHEA-C*_*16*_*-iron oxide in cultured Chinese hamster lung cells*

There were no statistically significant increases in the frequencies of aberrant metaphases with structural or numerical aberrations in the PHEA-C_16_-iron oxide-treated groups compared with the corresponding negative control groups. In the positive control groups treated with benzo[a]pyrene (+S) or ethylmethanesulfonate (−S), positive responses were observed. Therefore, it was concluded that PHEA-C_16_-iron oxide did not induce chromosomal aberrations in the CHL cells which were used in this study.

## Discussion

In this study, we have prepared biocompatible PHEA-C_16_-iron oxide nanoparticles with sizes of 20, 70, and 150 nm, controlled by the amount of C_16_ as a hydrophobic side chain. *In vitro* and *in vivo* experiments were performed with 20-nm particles; USPIO was used because the purpose of this study was to develop a novel MRI contrast agent for the detection of lymph nodes. The PHEA-C_16_-iron oxide as compared with Resovist®, a clinically approved MRI contrast agent, showed slightly better imaging contrast in lower concentration of iron from the *in vitro* phantom test. The PHEA-C_16_-iron oxide could also effectively detect bone marrow and lymph node of the rabbit by MRI. A single-dose intravenous toxicity and gene toxicity study of the PHEA-C_16_-iron oxide was commissioned by the preclinical research center of ChemOn, Inc. The results of LD50 from single-dose toxicity test were calculated 1,500 mg/kg for males and 1,300 mg/kg for females. PHEA-C_16_-iron oxide was confirmed biocompatible and nontoxic because genetic toxicity tests show negative results. These results indicate the great potential applications of PHEA-C_16_-iron oxide for the detection of lymph nodes by MRI.

## Conclusions

The results suggest that the PHEA-C16-iron oxide has slightly better imaging contrast effect than the clinically approved contrast agent Resovist®. The PHEA-C16-iron oxide has excellent imaging contrast in the liver, blood vessel, and even in the lymph node. With its biocompatible and nontoxic characteristics, the PHEA-C16-iron oxide is a promising candidate for a new contrast agent.

## Competing interests

We certify that there is no conflict of interest with any financial organization regarding the material discussed in the manuscript.

## Authors’ contributions

As the main author, DCH conceived of the study and participated in its design and overall experiments. HYL drafted the manuscript and participated in the design of the study. YWS carried out the *in vitro* MRI experiments. BKK carried out the *in vivo* MRI experiments. BCS and SHC took part in the synthesis of the nanoparticle compounds. SHY carried out the NMR characterization analysis. YWC assisted with the toxicity experiments. CWC oversaw the study and provided insightful guidance. All authors read and approved the final manuscript.

## Authors’ information

DCH gained his MS degree in 2005 at the Department of Food Science, Kongju National University, Gongju, Republic of Korea. HYL attained his PhD degree in 2006 at the Department of Advanced Organic Materials Engineering, Chonbuk National University, Jeonju, Republic of Korea. YWS received her BS diploma in 2012 at the Department of Chemistry, Chungnam National University, Daejeon, Republic of Korea. SHY gained his PhD degree in 1987 at the Department of Physics and Chemistry, Korea Advanced Institute of Science and Technology, Daejeon, Republic of Korea. YWC attained his PhD degree in 2007 at the College of Pharmacy, Kangwon National University, Chuncheon, Republic of Korea. BKK gained his PhD degree in 2002 at the Department of Radiology, Chung-Ang University, Seoul, Republic of Korea. BCS achieved his PhD degree in 1998 at the Department of Physics and Chemistry, Korea Advanced Institute of Science and Technology, Daejeon, Republic of Korea. CWC gained his PhD degree in 1996 at the College of Pharmacy, Chonnam National University, Kwangju, Republic of Korea. SHC received his PhD degree in 2000 at the Department of Physics and Chemistry, Korea Advanced Institute of Science and Technology, Daejeon, Republic of Korea.
